# Intravital Microscopy (IVM) in Human Solid Tumors: Novel Protocol to Examine Tumor-Associated Vessels

**DOI:** 10.2196/15677

**Published:** 2020-10-09

**Authors:** Denslow Allerton Trumbull, Riccardo Lemini, Sanjay P Bagaria, Enrique F Elli, Dorin T Colibaseanu, Michael B Wallace, Emmanuel Gabriel

**Affiliations:** 1 College of Medicine University of Florida Gainesville, FL United States; 2 Department of Surgery Mayo Clinic Jacksonville, FL United States; 3 Department of Gastroenterology Mayo Clinic Jacksonville, FL United States; 4 Division of Surgical Oncology Department of Surgery Mayo Clinic Jacksonville, FL United States

**Keywords:** intravital microscopy, solid tumors, microvasculature

## Abstract

**Background:**

Intravital microscopy (IVM) allows the real-time, direct visualization of microscopic blood vessels. This pilot clinical trial will elucidate the physical and functional characteristics of vessels associated with solid tumors.

**Objective:**

The main objective of this study is to determine the feasibility of performing IVM in patients with solid tumors during the standard course of surgical resection. IVM will also be performed when vasopressors or fluid boluses are administered during the standard course of the operation.

**Methods:**

This is an open-label, nonrandomized, single-center, pilot study of IVM observation in subjects with solid tumors undergoing surgical resection.

**Results:**

This study was active on January 1, 2019 (NCT03823144) and funded by the Mayo Clinic Florida Cancer Focused Research Team Award. As of September 27, 2020, we had enrolled 20 patients. Accrual period is expected to end by December 31, 2021.

**Conclusions:**

This trial will support the development of interventions to improve patient treatment by extending the application of IVM to the tumor microenvironment. IVM observations during volume and pressor management at the time of surgery may aid in the development of strategies to augment responses to systemic treatments.

**International Registered Report Identifier (IRRID):**

PRR1-10.2196/15677

## Introduction

Intravital microscopy (IVM) is the microscopic observation of living tissue in real-time. IVM has been used to show that tumor vessels lack the sequential hierarchy of normal vessels such that arterioles, capillaries, and venules typically cannot be discriminated within tumor tissues [1,2]. This disorganization of aberrant tumor vessels was demonstrated in a previous clinical trial [3]. These tumor-associated vessels were also characterized by irregular diameters, aberrant branching patterns, abnormal blood flow rates, and anastomotic strictures. These characteristics could have profound influence on the delivery of agents (ie, chemotherapy or cellular immunotherapy) to the tumor microenvironment [4].

Human IVM was used first in the investigation of melanoma primary tumors. IVM has also been used to directly examine the hemodynamic properties of tumor vessels in preclinical mouse models [5-8]. Recent studies in B16 murine melanoma have used IVM to demonstrate that the abnormal, tortuous vascular structure in tumors represents a bottleneck to adoptive cell transfer immunotherapy because of poor trafficking of cytotoxic effector T cells at this site [5]. Others have used different forms of IVM to observe lymphatics or metastatic disease in real-time in mouse models [9,10]. There has also been reported real-time imaging of human tumor and lymphatics using different techniques such as multiphoton imaging, high-resolution ultrasound, or optical coherence tomography [11,12].

Our trial has the strong potential to expand upon novel tumor imaging techniques and not only form a directly observed basis of the tumor vasculature as a barrier to systemic drug efficacy in humans but also establish a rationale to overcome this barrier. To this end, this trial has the following objectives.

### Objectives

#### Primary Objective – Part I (10 Patients)

The main objective is to determine the feasibility of performing IVM in patients with deep space solid tumors during the standard course of surgical treatment (resection). A successful intravital microscopic observation will include the ability to complete each of the following:

1. Identify and measure vessels associated with tumor.
2. Determine vessel density per 10x field.
3. Visualize vital dyes within the vessels (fluorescein).
4. Calculate the blood flow velocity of the vessels and tissue penetration of fluorescein as a marker of vessel permeability.

#### Secondary Objective – Part II (40 Patients)

To assess the secondary objectives, we will:

1. Compare the microscopic observation of the tumor-associated vessels with normal tissue (peritoneal surface) in each individual subject.
2. Correlate the microscopic observations of the tumor-associated vessels with pathologic grade of tumor.
3. Correlate the microscopic observation of the microvasculature with tumor-specific and overall survival.

### Primary and Secondary Endpoints

#### Primary Endpoint

A patient observation will be deemed a success if each of the following parameters was measured:

1. Identify tumor-associated vessels and measure vessel diameters.
2. Determine vessel density per 10x field.
3. Visualize vital dyes within the tumor-associated vessels (fluorescein).
4. Calculate the blood flow velocity of the tumor-associated vessels and tissue penetration of fluorescein as a marker of vessel permeability.

Measurements will be obtained before and after any tumor vessel manipulation with either bolus fluids or vasopressors.

#### Secondary Endpoints

Measurements to assess the secondary endpoints include the following:

1. Postoperative comparison of the microvasculature of tumor with normal tissue (eg, peritoneum) in each individual subject using vessel diameters, vessel density, detection of intravital dye, and flow rates.
2. Postoperative correlation of the microvasculature with pathologic features of the tumor (ie, tumor grade) at the time of the final pathology report (5-7 days after surgery).
3. Postoperative correlation of the microscopic observation of the tumor microvasculature tumor-specific and overall survival.

## Methods

### Study Design

This is an open-label, nonrandomized, single-center study of IVM observation in conjunction with fluorescein in subjects with deep space solid tumors undergoing surgical resection. The first part is a pilot study of feasibility. Subjects will be treated on an inpatient basis.

### Study Protocol

Surgical resection will be performed as part of standard of care. Approach may include open, laparoscopic, or robotic, as the IVM microscope can be used through any of these approaches.

The IVM technology consists of a high-resolution confocal endomicroscope (Cell Vizio) supplied by Mauna Kea Technologies. This apparatus has the ability to provide single cell resolution and high-quality images of the microvasculature. While the typical application of this device is to investigate gastrointestinal mucosal surfaces (ie, esophagus or colon), it can be easily applied to any surface. It can be sterilized via standard STERRAD techniques or used with a sterile sleeve in order to interface with the peritoneal surface, as currently used in NCT03517852: Intravital Microscopy (IVM) in Patients with Peritoneal Carcinomatosis (PC) [13].

The microscope is positioned in the operating room table by the operating surgeon after the tumor is exposed. Once the microscope is in the proper position, the epifluorescent light source is turned on, and digital video recording commences. Then, 1-2 mL of 25% fluorescein is injected intravenously, and observation continues until loss of fluorescence is observed over 2-4 areas of both grossly normal and tumor tissue. The fluorescein is almost immediately visible within the microscopic field, and the vessels are quickly outlined in great detail. The fluorescence lasts for a few minutes (2-3 minutes) and then either fades or begins to permeate through the tissue. The extravasation of dye will be determined by visualizing fluorescence outside of the defined vessels for a total time of approximately 5 minutes. On average, the total obervation time per field is 1-2 minutes.

During the course of the surgery, if fluid boluses or vasopressors (eg, vasopressin or phenylephrine) are required and administered to maintain blood pressure during the resection, another round of IVM observations will be performed with a second dose of 1-2 mL of fluorescein. Communication with anesthesia will indicate when fluid boluses or vasopressors are administered, in order to coordinate the observation.

Technical difficulties with the microscope apparatus (eg, malfunction of the software or structural damage to any of the microscope components) or unforeseen events during the course of surgery that are unrelated to the study intervention but that result in the termination of the surgery (eg, adverse reaction to anesthetic prior to administration of fluorescein or hemodynamic instability from a complication of the surgery) will not be considered failures of the primary objective. If vasopressors are not administered during surgery, the patient data will still be included in the study as they address the primary objective, which is to determine the feasibility of IVM.

Offline analysis of digitally recorded live video will be performed using parameters and statistical methods that have been developed in our preclinical imaging studies [5]. Lumenal cross-sectional diameter (D) of vessels and velocity (V) of dye-labeled cells will be measured in offline observations. Wall shear rate (γ) will be calculated as 8(V/D) [14]. Vessel density will be determined by measuring the calculated blood vessel area as a percent of the total visual field area. If visible, the uptake of fluorescein will be measured as a diffusion rate (distance from tumor vessel over time) and as a percent of the total tumor field observed (percent of visual field expressed as surface area with dye detected). Blood flow velocity will be determined by the equation Q = (red blood cell velocity/1.6) x (d/2) x pi.

#### Target Accrual and Study Duration

A maximum of 50 subjects (Part I, 10 patients; Part II, 40 patients) will be enrolled. The number of subjects required is a function of the expected feasibility. Accrual is expected to take up to 2 years.

#### Inclusion Criteria

Patients who are aged ≥18 years will be included in this trial. Additionally, these patients must have an ECOG Performance ≤2. Patients with a measurable tumor by direct visualization (visible lesion typically >0.5 cm in maximal diameter) and deep space tumors that meet indications for resection also require a negative skin-prick test to fluorescein. Tumors can be benign or malignant.

#### Exclusion Criteria

Patients who are experiencing uncontrolled intercurrent illnesses including, but not limited to, ongoing or active infection, symptomatic congestive heart failure, unstable angina pectoris, cardiac arrhythmia, psychiatric illness, or social situations will be excluded. Other factors for exclusion include renal dysfunction, as defined as a glomerular filtration rate <45; liver dysfunction, as defined by Child-Pugh score >5 or liver function tests 1.5 times above the normal average; any known allergy or allergic reaction to fluorescein or positive skin-prick test to fluorescein; and pregnant or nursing female subjects.

### Study Procedure

#### Baseline Evaluations

**Figure 1:**
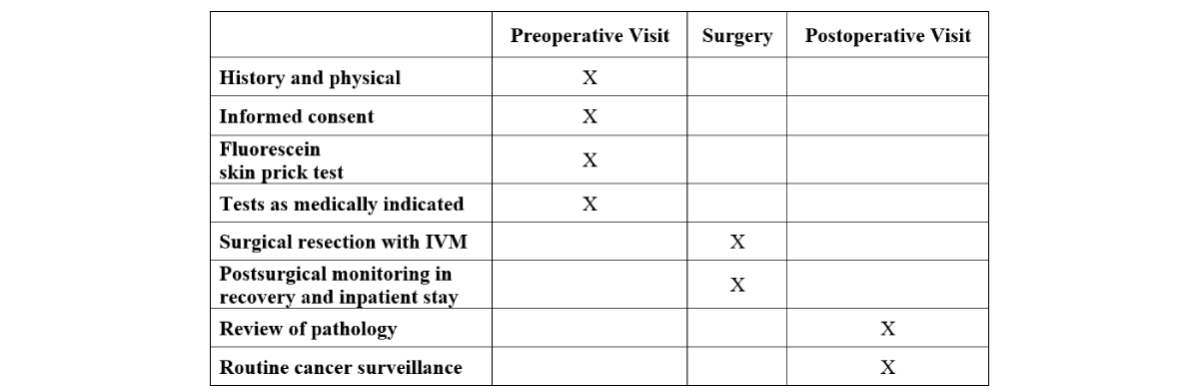
Summary of the evaluations performed during the protocol. IVM: intravital microscopy.

The standard presurgical assessment with labs and studies as determined by the preoperative anesthesia clinic will be used. A summary of the evaluations that should be performed during the preoperative visit, surgery, and postoperative visit is provided in Figure 1.

If determined to be eligible for the study and after giving informed consent, a skin-prick test to determine sensitivity and risk of anaphylaxis to fluorescein will be performed by the principal investigator. For testing allergic response to fluorescein, a sterile Duotip® lancet (Lincoln Medical, Decatur, IL) is used in 2 separate areas of skin on the forearm. The skin pricks will include approximately 4 µL of 25% fluorescein administered in areas of skin prepped with an alcohol pad. After 30 minutes, the appearance of a wheal greater than 3 mm in diameter is considered a positive test result. If a positive test is noted, the patient will no longer be eligible for this study. For comfort and relief of any skin irritation or pruritis, the patient will be offered an antihistamine (one 30-mg fexofenadine [Allegra] by mouth) following the skin-prick test if necessary.

#### Posttreatment Follow-Up Evaluations

The standard follow-up and safety evaluations from surgery includes a postoperative visit at 2-3 weeks and scheduled follow-up based upon final staging of the patient’s cancer. Follow-up will be based upon current National Comprehensive Cancer Network guidelines.

At the completion of the surgery, if gross tumor is entirely debulked (R0 resection), then patients are considered disease-free. Treatment response will be based upon the presence of a recurrence of tumor at either the primary site, locoregional recurrence (peritoneum), or metastatic sites.

The time to recurrence will determine the standard length of clinical follow-up. However, for this study, a 10-year limit will be placed on clinical data collection during the standard clinical follow-up. After a patient is enrolled, the duration of data collection will end at 10 years from the time of microscopic observation (surgery). These data (time of recurrence or survival) will be correlated with findings from the one-time, initial IVM observation for the defined 10-year period of data collection.

## Results

This study was active on January 1, 2019 (NCT03823144) and funded by the Mayo Clinic Florida Cancer Focused Research Team Award. As of September 27, 2020, we had enrolled 20 patients. Accrual period is expected to end by December 31, 2021.

## Discussion

We anticipate that this study will elucidate the structural and hemodynamic properties of vessels associated with solid tumors, building on previous studies that used IVM in human cancers [3,15]. In order to model the effects of tumor-associated vessel function on patient outcomes, direct examination of the microvasculature is essential and critical to be performed in humans. Thus, the significance of our trial lies in the opportunity to develop interventions that improve patient care. Specifically, the detection of blood flow parameters (including vessel diameter, flow rates, vessel density, and fluorescent markers of tissue diffusion) in human solid tumors is expected to have utility in predicting clinical response to systemically delivered therapies. This is because, in order for effective intravenous therapies to reach target tumors, they must have functional tumor-associated vessels to travel through. Therefore, it is anticipated that the investigation of tumor-associated vessels in real-time through IVM will lead to a better understanding of factors influencing systemic drug efficacy and perhaps generate a more complete picture of the tumor-associated locoregional vasculature.

In addition, by adding IVM observations during the course of volume and pressor management during the course of surgery, further data regarding tumor vessel dynamics will be obtained, which may offer a means to augment responses to systemic treatments. Ongoing studies by our group are investigating whether tumor-associated vessels can be manipulated in order to augment drug delivery. However, the first step in reaching this innovative goal is to establish the feasibility and safety of IVM in human solid tumors, which is the primary objective of this trial.

Indeed, novel approaches using IVM have the potential to provide advances in the field of personalized medicine by identifying patients who may respond to systemically delivered chemotherapeutic drugs or immunotherapeutic agents. IVM has been applied to the endoscopic evaluation of gastrointestinal tumors, the cystoscopic evaluation of bladder urothelial cancer, and most recently to melanoma [3]. The superficial or endoluminal location of these tumor types facilitates IVM, and from these studies, advances have been made in the understanding of tumor vasculature and the diagnosis of endoluminal cancers [15]. Our current trial will take these studies another step further through investigation of deep space solid tumors, both benign and malignant.

We anticipate completion of trial enrollment by December 31, 2020. IVM results will be correlated with oncologic outcomes. While the microscope is used to observe tumor-associated vessels, the procedure is not anticipated to cause iatrogenic spread of tumors. This is because the microscope does not puncture the tumors but is only used to examine the tumor-associated vessels. Furthermore, digital and instrument manipulation, both blunt and sharp, is used to resect the tumor, which provides more force than the microscope. In addition, this study may be limited in that mainly surface tumor vessels will be observed, whereas deeper vessels will not be observed. Observation of deeper vessels will not be performed as this would require incision into the tumor, which is contraindicated.

In conclusion, our trial will support the development of interventions to improve patient treatment by extending the application of IVM to the tumor microenvironment. IVM observations during volume and pressor management at the time of surgery may also aid in the development of strategies to augment responses to systemic treatments.

## Abbreviations

D: cross-sectional diameter
IVM: intravital microscopy
V: velocity
